# Nonlinear relationship between untraditional lipid parameters and the risk of prediabetes: a large retrospective study based on Chinese adults

**DOI:** 10.1186/s12933-023-02103-z

**Published:** 2024-01-06

**Authors:** Mingkang Li, Wenkang Zhang, Minhao Zhang, Linqing Li, Dong Wang, Gaoliang Yan, Yong Qiao, Chengchun Tang

**Affiliations:** Department of Cardiology, Zhongda Hospital, Southeast University, 87 Dingjiaqiao, Nanjing, 210009 Jiangsu China

**Keywords:** Untraditional lipid parameters, Prediabetes, Nonlinearly, Chinese adults

## Abstract

**Background:**

Abnormal lipid metabolism poses a risk for prediabetes. However, research on lipid parameters used to predict the risk of prediabetes is scarce, and the significance of traditional and untraditional lipid parameters remains unexplored in prediabetes. This study aimed to comprehensively evaluate the association between 12 lipid parameters and prediabetes and their diagnostic value.

**Methods:**

This cross-sectional study included data from 100,309 Chinese adults with normal baseline blood glucose levels. New onset of prediabetes was the outcome of concern. Untraditional lipid parameters were derived from traditional lipid parameters. Multivariate logistic regression and smooth curve fitting were used to examine the nonlinear relationship between lipid parameters and prediabetes. A two-piecewise linear regression model was used to identify the critical points of lipid parameters influencing the risk of prediabetes. The areas under the receiver operating characteristic curve estimated the predictive value of the lipid parameters.

**Results:**

A total of 12,352 participants (12.31%) were newly diagnosed with prediabetes. Following adjustments for confounding covariables, high-density lipoprotein cholesterol (HDL-C) and low-density lipoprotein cholesterol were negatively correlated with prediabetes risk. Conversely, total cholesterol, triglyceride (TG), lipoprotein combine index (LCI), atherogenic index of plasma (AIP), non-HDL-C, atherogenic coefficient, Castelli’s index-I, remnant cholesterol (RC), and RC/HDL-C ratio displayed positive correlations. In younger adults, females, individuals with a family history of diabetes, and non-obese individuals, LCI, TG, and AIP exhibited higher predictive values for the onset of prediabetes compared to other lipid profiles.

**Conclusion:**

Nonlinear associations were observed between untraditional lipid parameters and the risk of prediabetes. The predictive value of untraditional lipid parameters for prediabetes surpassed that of traditional lipid parameters, with LCI emerging as the most effective predictor for prediabetes.

**Supplementary Information:**

The online version contains supplementary material available at 10.1186/s12933-023-02103-z.

## Background

Diabetes has emerged as the most prevalent and clinically significant metabolic disorder in recent decades, affecting over 536.6 million individuals globally in 2021. This widespread impact places a substantial burden on both public health and healthcare expenditures [1]. Prediabetes, an intermediary state of hyperglycemia between normal blood glucose and diabetes, manifests before the onset of diabetes. According to the 2018 American Diabetes Association (ADA) diagnostic criteria, the prevalence of prediabetes among Chinese adults is 35.2% [2]. Without prompt management, the annual rate of conversion to diabetes is 5% ~ 10% [3]. Moreover, prediabetes serves as a pivotal warning sign, indicating a heightened risk for future cardiovascular and cerebrovascular disorders, microvascular diseases, cancers, dementia, and other diseases [4–6]. Hence, timely intervention and effective management strategies among populations with prediabetes are crucial in preventing abnormal progression and complications associated with glucose metabolism.

Abnormal lipid metabolism significantly contributes to prediabetes. Dyslipidemia-induced lipotoxicity plays a crucial role in two primary pathways to prediabetes: peripheral insulin resistance (IR) and pancreatic islet β cell dysfunction [7]. Excessive cholesterol accumulation impairs β cell function, disrupting glucose tolerance and insulin secretion. Assessments such as the hyperinsulinemic-euglycemic clamp test and homeostatic model measure IR and diabetes risk but pose challenges due to the time, expense, complexity, and invasiveness [8, 9]. Patients with prediabetes often exhibit quantitative lipoprotein, qualitative lipoprotein, and kinetic abnormalities, fostering a shift to a more atherogenic lipid profile, including higher total cholesterol (TC), triglyceride (TG), and low-density lipoprotein (LDL) cholesterol (LDL-C) levels, and lower high-density lipoprotein cholesterol (HDL-C) [10]. Recent studies highlight untraditional lipid indicators such as non-HDL-C, remnant cholesterol (RC), and RC/HDL-C ratio, derived from multiple traditional lipid parameters, as alternatives for IR. These markers relate closely to diabetes and cardiovascular and cerebrovascular diseases [11–13]. Compared to traditional lipid parameters, these untraditional parameters offer richer insights, quantifying risk information and balancing atherogenic and anti-atherogenic lipoproteins more effectively [14].

However, the relationship between untraditional lipid parameters and prediabetes remains unknown. It is unclear which of these parameters is most effective in detecting prediabetes. Therefore, a comprehensive comparative analysis of the relationship between untraditional lipid parameters and the prevalence of prediabetes in the Chinese adult population was conducted using nationally representative large-scale research data for this study.

## Methods

### Data source and study participants

This study sourced its data from the Dryad public database (https://datadryad.org/stash/dataset/doi:10.5061%2Fdryad.ft8750v), originally provided by Chen et al. [15]. This dataset comprised medical data from 211,833 individuals who underwent health examinations at Rich Healthcare Group across 32 sites and 11 cities in China from 2010 to 2016. According to the Dryad database terms, the dataset can be used for secondary analysis to explore new research hypotheses and optimize data utilization.

**Fig. 1:**
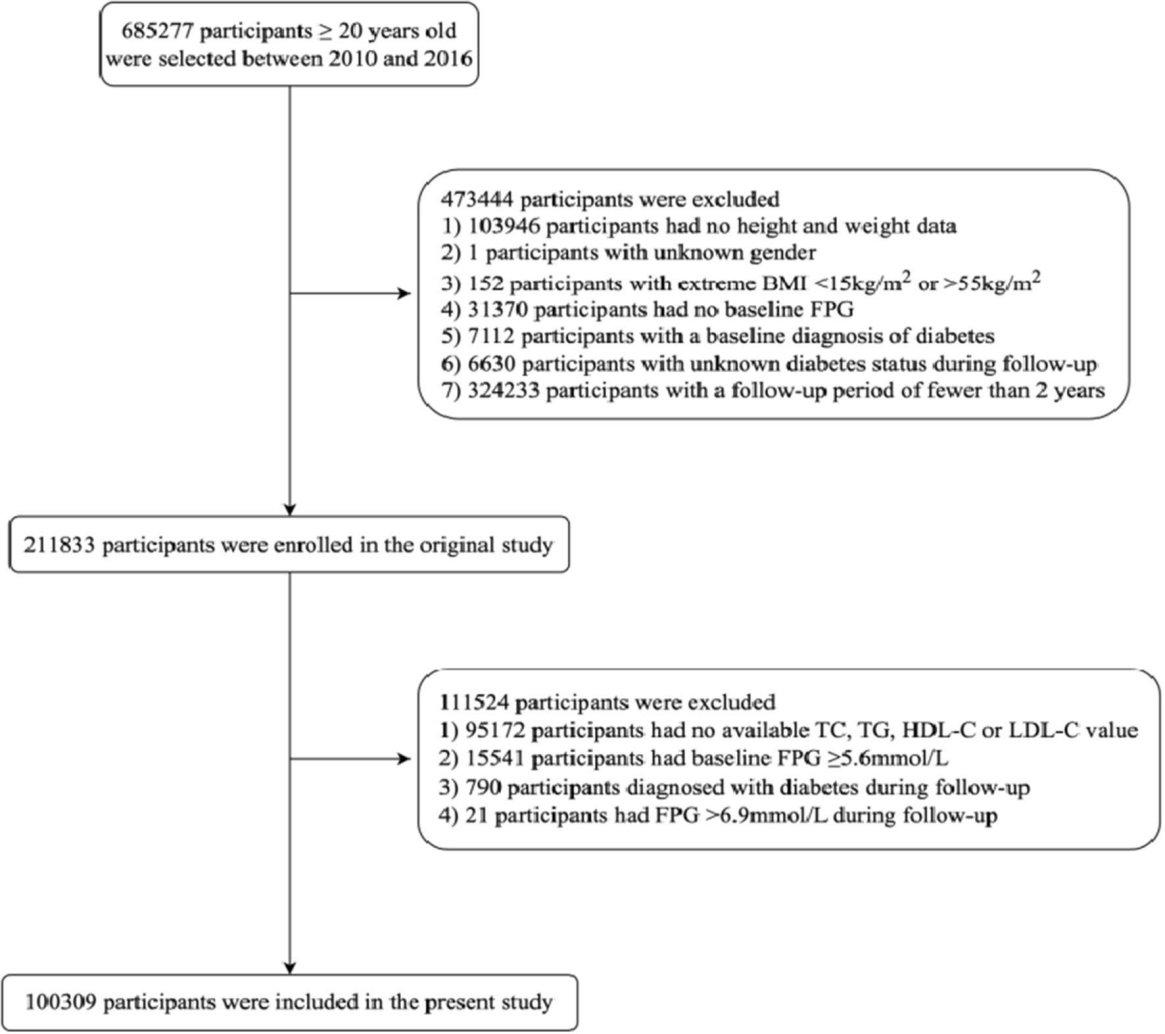
Flow diagram of the participants selection

The original study recruited 685,277 Chinese adults over 20 years old, with at least two visits from 2010 to 2016. Exclusion criteria were as follows: (1) missing height and weight data (n = 103,946); (2) unknown gender (n = 1); (3) extreme body mass index (BMI), defined as a BMI of < 15 kg/m^2^ or > 55 kg/m^2^ (n = 152); (4) missing baseline fasting plasma glucose (FPG) data (n = 31,370); (5) baseline diabetes (n = 7112); (6) unknown diabetes status during follow-up (n = 6630); (7) had a follow-up period of less than 2 years (n = 324,233). Ultimately, 211,833 participants were enrolled in the original study. This study, following Chen et al., endeavors to further investigate the relationship between untraditional lipid parameters and prediabetes. Additional exclusion criteria based on the ADA’s prediabetes diagnostic criteria: (1) no data on TC, TG, HDL-C, or LDL-C (n = 95,172); (2) baseline FPG ≥ 5.6 mmol/L (n = 15,541); (3) diabetes diagnosis during follow-up (n = 790); (4) FPG > 6.9 mmol/L during follow-up (n = 21). Finally, 100,309 participants were included in the current research (Fig. 1). This study received an ethical exemption from the Ethics Committee of Southeast University Affiliated Zhongda Hospital, complying with Dryad’s publication criteria.

### Data collection

Investigators obtained sociodemographic data from participants using standardized questionnaires, covering age, gender, smoking and drinking habits, and family history of diabetes. Blood pressure was measured by trained personnel using a mercury sphygmomanometer at rest. Smoking and drinking status were classified into four categories based on the baseline visit time: never, once, current, and unrecorded. Height and weight were measured by staff without shoes and heavy clothing. BMI was calculated as weight (kg)/height^2^ (m^2^).

Professional healthcare workers obtained fasting venous blood samples from participants after a minimum 10-h fast every visit. TC, TG, LDL-C, HDL-C, alanine aminotransferase (ALT), aspartate aminotransferase (AST), FPG, serum creatinine (Scr), and blood urea nitrogen (BUN) levels were measured using an automated analyzer (Beckman Coulter AU5800, Brea, CA, USA); The glucose oxidase method was used to measure FPG levels.

The metabolic score for IR (METS-IR), a novel index for insulin sensitivity, predicts visceral adiposity and incident diabetes [16]. The formula for calculating METS-IR was: ln [(2 × FPG) + TG] × BMI/[ln (HDL-C)]. Moreover, the Chinese diabetes risk score (CDRS), established by Ji et al. in 2013, is an effective non-invasive tool for prediabetes screening. The prediabetes screening strategy using CDRS gained expert consensus in 2023 [17]. The scoring rules of CDRS are presented in Additional file 1: Table S1.

The untraditional lipid parameters were calculated as follows:

Lipoprotein combine index (LCI) = TC × TG × LDL-C/HDL-C [18];

Atherogenic index of plasma (AIP) = lg (TG/HDL-C) [19];

Non-HDL-C = TC − HDL-C [20];

Atherogenic coefficient (AC) = non-HDL-C/HDL-C [14];

Castelli’s index-I (CRI-I) = TC/HDL-C [21];

Castelli’s index-II (CRI-II) = LDL-C/HDL-C [21];

RC = TC − HDL-C − LDL-C [22];

RC/HDL-C ratio = RC/HDL-C.

## Definitions

According to the 2018 ADA diagnostic criteria, prediabetes was defined as patients who did not develop diabetes throughout the follow-up period but had an FPG level between 5.6 and 6.9 mmol/L [23].

### Statistics analysis

Data analysis was performed using R version 4.2.0 (R Foundation), EmpowerStats (http://www.empowerstats.com, X&Y Solutions, Inc., Boston, MA), and GraphPad Prism (version 9.1.1 for macOS, GraphPad Software, LLC). Normally distributed continuous variables are expressed as the mean ± standard deviation, while skewed continuous variables are expressed as the median (25th to 75th interquartile range). Between-group differences were compared using t-tests or rank sum tests. Categorical variables are presented as frequencies with percentages, and comparisons were made using chi-square or Fisher’s exact test. The research hypothesis was tested through a series of analytical steps.

Initially, missing values in the dataset were addressed using specific methods. For continuous variables such as ALT, AST, Scr, systolic blood pressure (SBP), and diastolic blood pressure (DBP), mean or median imputation was applied. Smoking and drinking status, categorized as group-based variables, treated their missing values collectively (labelled as unrecorded).

Next, the correlation between lipid parameters, METS-IR, and CDRS was evaluated using Pearson or Spearman’s rank correlation coefficient analyses. Collinearity analysis was performed to calculate the covariate’s variance inflation factor (VIF). Covariates exhibiting a VIF of > 5 were considered collinear and consequently excluded from subsequent multivariate logistic regression models.

Next, a univariate logistic regression model was utilized to assess each variable's influence on prediabetes risk, recording the odds ratio (OR) and corresponding 95% confidence interval (CI). Following guidelines from the Strengthening the Reporting of Observational Studies in Epidemiology statement, three epidemiological-based multivariate logistic regression models were constructed. Model 1 was adjusted for baseline age and gender, while Model 2 additionally considered a family history of diabetes, BMI, SBP, and DBP in addition to Model 1. Model 3 encompassed all noncollinear variables. Lipid parameters were transformed into quartiles, forming the basis for the final model to ensure result reliability. This model evaluated the relationship between the quartiles and prediabetes, taking the lowest quartile as the reference. Furthermore, a generalized additive model with a fitting smoothness was employed to delineate the dose–response relationship between lipid parameters and prediabetes risk. Additionally, a two-piecewise logistic regression model was constructed to uncover potential hidden nonlinear relationships by analyzing data on both sides of the inflexion point. The log-likelihood ratio aided in selecting the most appropriate model characterizing the relationship between lipid parameters and prediabetes risk.

Additionally, stratified analyses were conducted based on Model 3 to investigate other factors influencing the relationship between lipid parameters and the onset of prediabetes. Stratification occurred according to age (< 60 years, ≥ 60 years), gender (male, female), BMI (< 24 kg/m^2^, ≥ 24 kg/m^2^), and family history of diabetes (yes, no).

Subsequently, receiver operating characteristic (ROC) curves were constructed to estimate each lipid parameter’s predictive ability and accuracy for prediabetes risk and determine the optimal cut-off values. These analyses were further refined based on the stratification above, calculating the area under the ROC curve (AUC) for each subgroup to identify the most effective lipid parameter in predicting prediabetes. All tests were two-tailed, and P < 0.05 was considered statistically significant.

## Results

### Baseline characteristics of participants

In this study, 100,309 participants without prediabetes at baseline had an average age of 42.91 ± 12.45 years, with males comprising 51.97% of the cohort. Among them, 12,352 participants (12.31%) developed prediabetes during an average observation period of 37.4 months. Table 1 delineates the baseline characteristics of the study population, categorized by prediabetes diagnosis. Participants in the prediabetes group were more likely to be older, males, current smokers, and current drinkers, with a higher prevalence of family history of diabetes. Moreover, compared to those without prediabetes, individuals with prediabetes exhibited elevated BMI, SBP, DBP, FPG, ALT, AST, Scr, BUN, TC, TG, LDL-C, LCI, AIP, non-HDL- C, AC, CRI-I, CRI-II, RC, RC/HDL-C ratio, METS-IR, and CDRS levels, alongside lower HDL-C levels (all P < 0.05).

**Table 1:** Baseline characteristics of participants with and without prediabetes

Variables	Overall	Non-prediabetes	Prediabetes	P value
Participants	100,309	87,957	12,352	
Age, years	42.91 ± 12.45	42.05 ± 12.04	49.00 ± 13.59	< 0.001
Male, n (%)	52,130 (51.97)	44,395 (50.47)	7735 (62.62)	< 0.001
Smoking status, n (%)				< 0.001
Current	5349 (5.33)	4509 (5.13)	840 (6.80)	
Once	1090 (1.09)	935 (1.06)	155 (1.25)	
Never	21,199 (21.13)	18,877 (21.46)	2322 (18.80)	
Not recorded	72,671 (72.45)	63,636 (72.35)	9035 (73.15)	
Drinking status, n (%)				< 0.001
Current	639 (0.64)	539 (0.61)	100 (0.81)	
Once	4548 (4.53)	3944 (4.48)	604 (4.89)	
Never	22,451 (22.38)	19,838 (22.55)	2613 (21.15)	
Not recorded	72,671 (72.45)	63,636 (72.35)	9035 (73.15)	
Family history of diabetes, n (%)	2208 (2.20)	1898 (2.16)	310 (2.51)	0.013
BMI, kg/m^2^	23.10 ± 3.22	22.91 ± 3.17	24.38 ± 3.25	< 0.001
SBP, mmHg	118.08 ± 16.08	117.08 ± 15.61	125.17 ± 17.51	< 0.001
DBP, mmHg	73.76 ± 10.77	73.21 ± 10.57	77.61 ± 11.38	< 0.001
FPG, mmol/L	4.79 ± 0.47	4.75 ± 0.47	5.03 ± 0.40	< 0.001
ALT, U/L	17.80 (12.80,26.50)	17.20 (12.50,26.00)	21.00 (14.70,31.00)	< 0.001
AST, U/L	23.71 (23.00,23.71)	23.71 (23.00,23.71)	23.71 (23.00,23.71)	< 0.001
Scr, umol/L	69.90 ± 15.64	69.44 ± 15.60	73.17 ± 15.52	< 0.001
BUN, mmol/L	4.63 ± 1.15	4.61 ± 1.14	4.84 ± 1.15	< 0.001
TC, mmol/L	4.75 ± 0.88	4.72 ± 0.88	4.92 ± 0.90	< 0.001
TG, mmol/L	1.06 (0.74,1.58)	1.02 (0.72,1.52)	1.30 (0.90,1.94)	< 0.001
HDL-C, mmol/L	1.38 ± 0.30	1.39 ± 0.31	1.34 ± 0.29	< 0.001
LDL-C, mmol/L	2.74 ± 0.67	2.73 ± 0.67	2.84 ± 0.67	< 0.001
LCI	9.72 (5.45,18.28)	9.28 (5.27,17.40)	13.62 (7.44,24.44)	< 0.001
AIP	-0.11(-0.30,0.10)	−0.12(−0.31,0.08)	−0.003(−0.20,0.20)	< 0.001
Non-HDL- C	3.37 ± 0.86	3.34 ± 0.85	3.58 ± 0.87	< 0.001
AC	2.58 ± 0.99	2.55 ± 0.98	2.81 ± 1.02	< 0.001
CRI-I	3.58 ± 0.99	3.55 ± 0.98	3.81 ± 1.02	< 0.001
CRI-II	2.08 ± 0.70	2.06 ± 0.70	2.21 ± 0.71	< 0.001
RC	0.56 (0.35,0.84)	0.55 (0.34,0.82)	0.67 (0.44,0.96)	< 0.001
RC/HDL-C ratio	0.41 (0.24,0.67)	0.40 (0.23,0.65)	0.50 (0.31,0.78)	< 0.001
METS-IR	33.20 ± 6.37	32.81 ± 6.26	35.92 ± 6.41	< 0.001
CDRS	14 (9,19)	13 (9,18)	19 (14,24)	< 0.001

*BMI* body mass index, *SBP* systolic blood pressure, *DBP* diastolic blood pressure, *FPG* fasting plasma glucose, *ALT* alanine aminotransferase, *AST* aspartate aminotransferase, Scr serum creatinine, *BUN* blood urea nitrogen, *TC* total cholesterol, *TG* triglyceride, *HDL-*C high-density lipoprotein cholesterol, *LDL-C* low-density lipoprotein cholesterol, *LCI* lipoprotein combine index, *AIP* atherogenic index of plasma, *AC* atherogenic coefficient, *CRI-I* Castelli’s index-I, CRI-II Castelli’s index-II, RC remnant cholesterol, *METS-IR* metabolic score for insulin resistance, *CDRS* Chinese diabetes risk score

### The correlation between baseline lipid parameters, METS-IR, and CDRS

Table 2 displays Spearman and Pearson correlation analyses, illustrating associations between traditional and untraditional lipid parameters with METS-IR and CDRS. AIP exhibited stronger linear correlations with METS-IR compared to other lipid parameters (r = 0.728 for AIP; r = 0.143 for TC; r = 0.638 for TG; r = -0.569 for HDL-C; r = 0.150 for LDL-C; r = 0.644 for LCI; r = 0.348 for non-HDL-C; r = 0.604 for AC; r = 0.604 for CRI-I; r = 0.517 for CRI-II; r = 0.467 for RC; r = 0.555 for RC/HDL-C ratio). Conversely, LCI demonstrated a more robust linear correlation with CDRS relative to other lipid parameters (r = 0.449 for LCI; r = 0.284 for TC; r = 0.415 for TG; r = -0.141 for HDL-C; r = 0.256 for LDL-C; r = 0.398 for AIP; r = 0.360 for non-HDL-C; r = 0.311 for AC; r = 0.311 for CRI-I; r = 0.280 for CRI-II; r = 0.312 for RC; r = 0.305 for RC/HDL-C ratio).

**Table 2:** The correlation between baseline lipid parameters, METS-IR, and CDRS

Variables	METS-IR		CDRS	
	Correlation coefficient (r)	P value	Correlation coefficient (r)	P value
Traditional lipid parameters				
TC	0.143	< 0.001	0.284	< 0.001
TG	0.638	< 0.001	0.415	< 0.001
HDL-C	−0.569	< 0.001	−0.141	< 0.001
LDL-C	0.150	< 0.001	0.256	< 0.001
Untraditional lipid parameters				
LCI	0.644	< 0.001	0.449	< 0.001
AIP	0.728	< 0.001	0.398	< 0.001
Non-HDL- C	0.348	< 0.001	0.360	< 0.001
AC	0.604	< 0.001	0.311	< 0.001
CRI-I	0.604	< 0.001	0.311	< 0.001
CRI-II	0.517	< 0.001	0.280	< 0.001
RC	0.467	< 0.001	0.312	< 0.001
RC/HDL-C ratio	0.555	< 0.001	0.305	< 0.001

*TC* total cholesterol, *TG* triglyceride, *HDL-C* high-density lipoprotein cholesterol, *LDL-C* low-density lipoprotein cholesterol, *LCI* lipoprotein combine index, *AIP* atherogenic index of plasma, *AC* atherogenic coefficient, *CRI-I* Castelli’s index-I, *CRI-II* Castelli’s index-II, *RC* remnant cholesterol

### Relationship between baseline lipid parameters and prediabetes

Table 3 summarizes the univariate logistic regression results, aiding in covariate selection for subsequent multivariate regression analysis. BMI, SBP, DBP, FPG, ALT, AST, Scr, BUN, TC, TG, LDL-C, LCI, AIP, non-HDL-C, AC, CRI-I, CRI-II, RC, and RC/HDL-C ratio were risk factors for prediabetes. Among these parameters, AIP presented as the most significant risk factor associated with prediabetes (OR 3.452, 95% CI 3.242–3.678). Then, collinearity analysis (Additional file 2: Table S2) identified high collinearity degrees for the smoking and drinking status, indicated by VIF values of 8.393 and 8.433, respectively. Consequently, these variables were excluded as covariates in multivariate logistic regression analysis.

**Table 3:** Univariate logistic analysis for predicting prediabetes

Variables	Univariate analysis		
	OR	95%CI	P value
Age	1.041	1.039–1.042	< 0.001
Male	1.644	1.581–1.709	< 0.001
Smoking status			
Current	Reference		
Once	1.312	1.215–1.417	< 0.001
Never	1.168	0.984–1.386	0.076
Not recorded	0.866	0.825–0.909	< 0.001
Drinking status			
Current	Reference		
Once	1.307	1.054–1.619	0.015
Never	1.079	0.987–1.178	0.093
Not recorded	0.928	0.886–0.972	0.002
Family history of diabetes	1.167	1.034–1.318	0.013
BMI	1.143	1.137–1.149	< 0.001
SBP	1.029	1.028–1.030	< 0.001
DBP	1.036	1.035–1.038	< 0.001
FPG	4.784	4.547–5.034	< 0.001
ALT	1.007	1.007–1.008	< 0.001
AST	1.011	1.008–1.013	< 0.001
Scr	1.015	1.014–1.016	< 0.001
BUN	1.182	1.163–1.200	< 0.001
TC	1.273	1.247–1.299	< 0.001
TG	1.313	1.291–1.335	< 0.001
HDL-C	0.603	0.565–0.642	< 0.001
LDL-C	1.267	1.233–1.302	< 0.001
LCI	1.014	1.013–1.015	< 0.001
AIP	3.452	3.242–3.678	< 0.001
Non-HDL- C	1.359	1.331–1.387	< 0.001
AC	1.272	1.251–1.295	< 0.001
CRI-I	1.272	1.251–1.295	< 0.001
CRI-II	1.312	1.280–1.345	< 0.001
RC	1.943	1.865–2.024	< 0.001
RC/HDL-C ratio	1.763	1.694–1.835	< 0.001

*BMI* body mass index, *SBP* systolic blood pressure, *DBP* diastolic blood pressure, *FPG* fasting plasma glucose, *ALT* alanine aminotransferase, *AST* aspartate aminotransferase, *Scr* serum creatinine, *BUN* blood urea nitrogen, *TC* total cholesterol, *TG* triglyceride, *HDL-C* high-density lipoprotein cholesterol, *LDL-C* low-density lipoprotein cholesterol, *LCI* lipoprotein combine index, *AIP* atherogenic index of plasma, *AC* atherogenic coefficient, *CRI-I* Castelli’s index-I, *CRI-II* Castelli’s index-II, *RC* remnant cholesterol, *OR* odds ratio, *CI* confidence interval

Based on epidemiology, three multivariate logistic regression models were employed to evaluate the relationship between baseline lipid parameters and prediabetes risk (Table 4). The demographic-adjusted model (Model 1) demonstrated associations between all lipid parameters and prediabetes risk. However, upon further adjustment for family history of diabetes, BMI, SBP, and DBP (Model 2), the association between LDL-C and CRI-II with prediabetes risk became non-significant. After adjusting for all noncollinear variables except lipid parameters (Model 3), the association between TC (OR 1.016, 95% CI 0.993–1.040) and prediabetes in traditional lipid parameters disappeared. An increase in TG (OR 1.062, 95% CI 1.042–1.082) exhibited a positive correlation with prediabetes risk. Conversely, HDL-C (OR 0.892, 95% CI 0.831–0.958) and LDL-C (OR 0.953, 95% CI 0.925–0.982) seemed to potentially act as protective factors against prediabetes. All untraditional lipid parameters, except CRI-II, exhibited associations with prediabetes risk. Importantly, AIP continued to demonstrate the highest risk factor association with prediabetes (OR 1.326, 95% CI 1.227–1.433), followed by RC (OR 1.257, 95% CI 1.201–1.315).

**Table 4:** Multivariate logistic regression analyses for the associations between lipid parameters with prediabetes

	Model 1		Model 2		Model 3	
	OR (95%CI)	P value	OR (95%CI)	P value	OR (95%CI)	P value
TC	1.109 (1.085,1.133)	< 0.001	1.046 (1.023,1.070)	< 0.001	1.016 (0.993,1.040)	0.164
TG	1.187 (1.166,1.207)	< 0.001	1.090 (1.070,1.110)	< 0.001	1.062 (1.042,1.082)	< 0.001
HDL-C	0.735 (0.687,0.787)	< 0.001	0.909 (0.848,0.975)	0.007	0.892 (0.831,0.958)	0.002
LDL-C	1.065 (1.035,1.096)	< 0.001	0.996 (0.967,1.026)	0.793	0.953 (0.925,0.982)	0.002
LCI	1.008 (1.007,1.009)	< 0.001	1.003 (1.002,1.004)	< 0.001	1.002 (1.001,1.003)	< 0.001
AIP	2.214 (2.065,2.374)	< 0.001	1.462 (1.356,1.577)	< 0.001	1.326 (1.227,1.433)	< 0.001
Non-HDL- C	1.154 (1.128,1.179)	< 0.001	1.061 (1.037,1.086)	< 0.001	1.031 (1.006,1.055)	0.013
AC	1.126 (1.105,1.147)	< 0.001	1.039 (1.019,1.060)	< 0.001	1.027 (1.006,1.048)	0.010
CRI-I	1.126 (1.105,1.147)	< 0.001	1.039 (1.019,1.060)	< 0.001	1.027 (1.006,1.048)	0.010
CRI-II	1.113 (1.084,1.143)	< 0.001	1.007 (0.979,1.036)	0.629	0.984 (0.956,1.013)	0.274
RC	1.498 (1.435,1.563)	< 0.001	1.271 (1.215,1.329)	< 0.001	1.257 (1.201,1.315)	< 0.001
RC/HDL-C ratio	1.397 (1.340,1.457)	< 0.001	1.188 (1.138,1.241)	< 0.001	1.186 (1.134,1.239)	< 0.001

Model 1: adjusted for age and gender at baseline

Model 2: further adjusted for family history of diabetes, BMI, SBP, and DBP based on model 1

Model 3: further adjusted for FPG, ALT, AST, Scr, and BUN based on model 2

*TC* total cholesterol, *TG* triglyceride, *HDL-C* high-density lipoprotein cholesterol, *LDL-C* low-density lipoprotein cholesterol, *LCI* lipoprotein combine index, *AIP* atherogenic index of plasma, *AC* atherogenic coefficient, *CRI-I* Castelli’s index-I, *CRI-II* Castelli’s index-II, *RC* remnant cholesterol, *OR* odds ratio, *CI* confidence interval

**Fig. 2:**
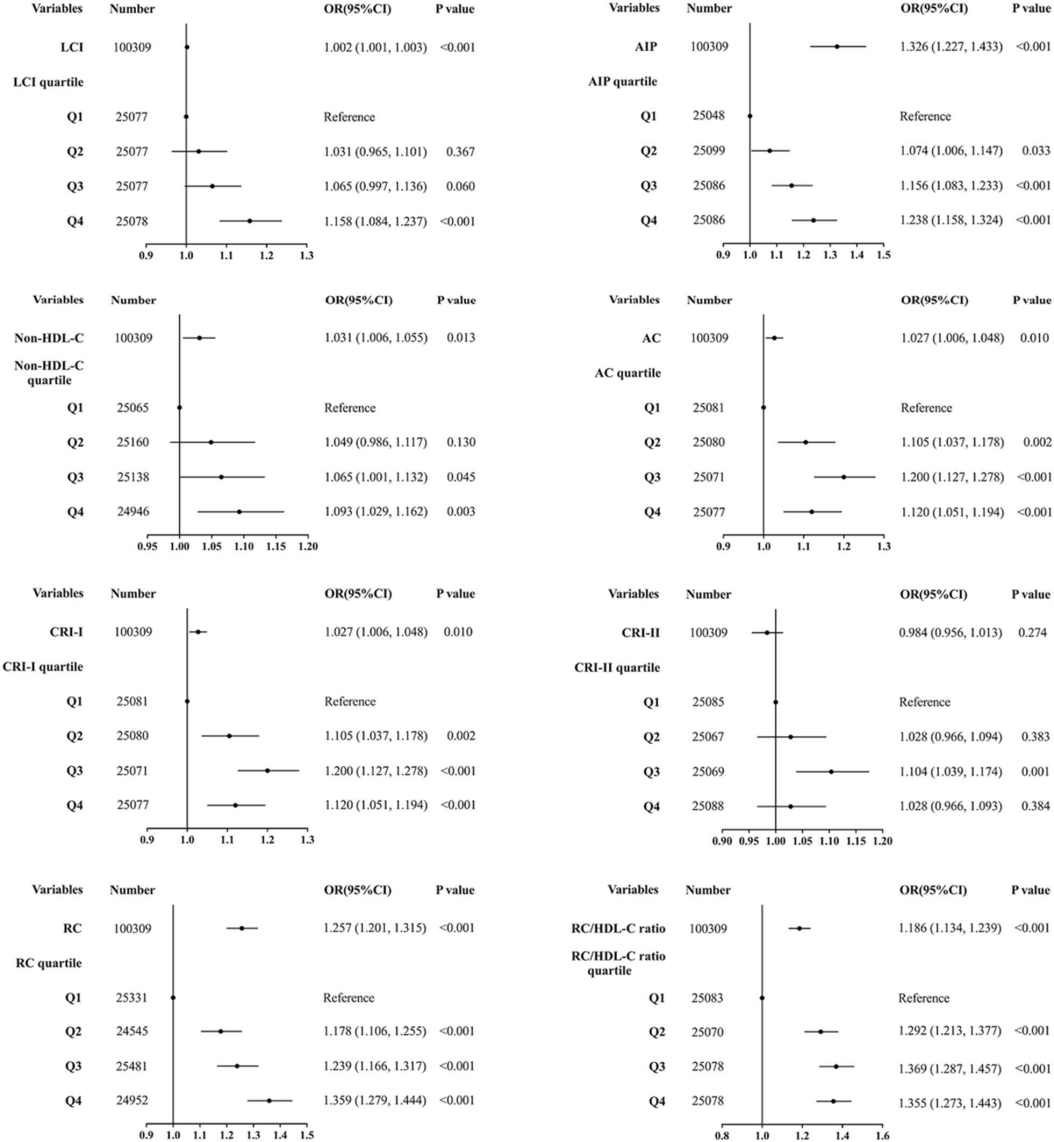
Forest plot of multivariate logistic regression analysis based on the quartile of untraditional lipid parameters in Model 3

**Fig. 3:**
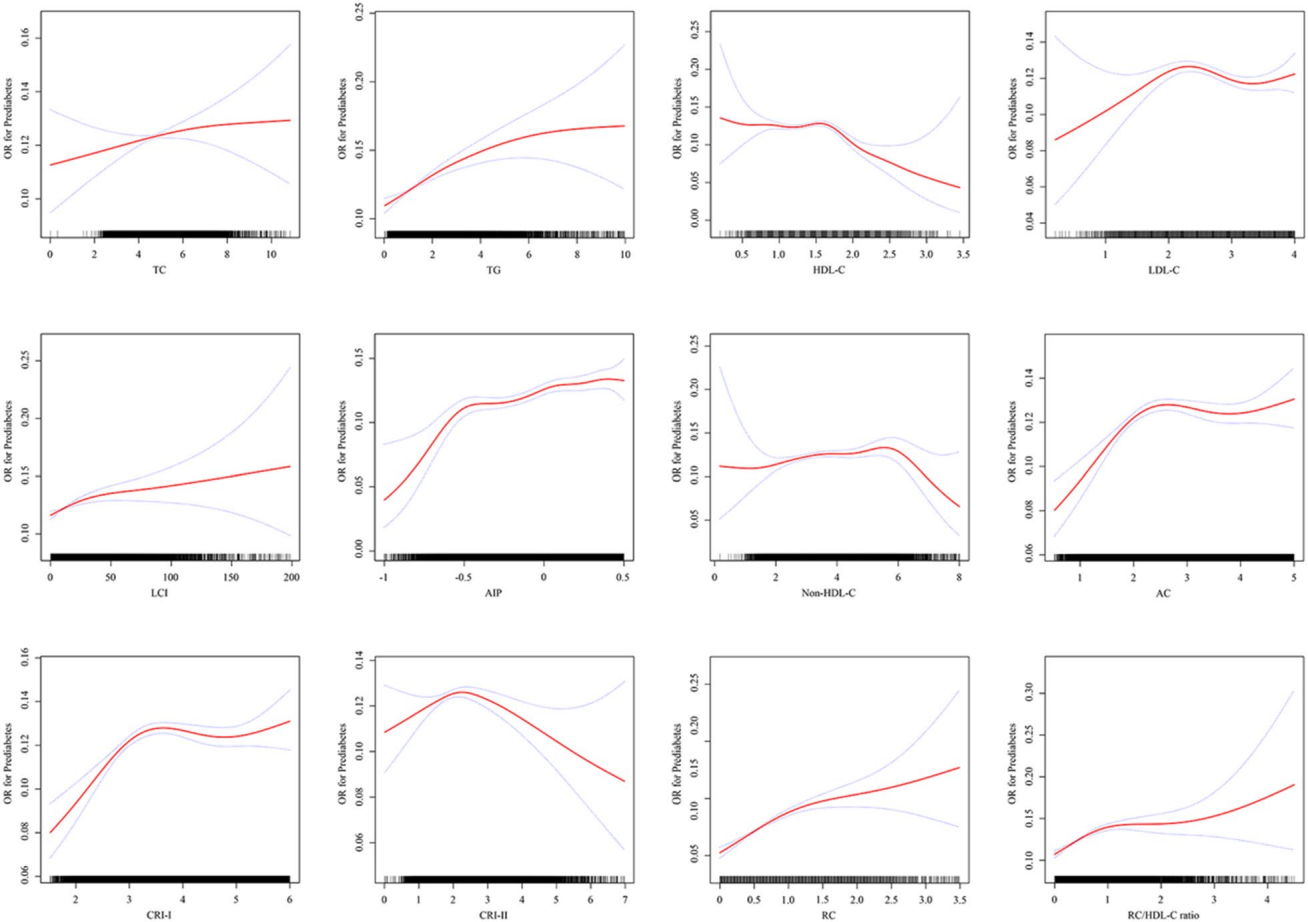
Generalized additive model with fitting smoothness for the dose–response relationship between lipid parameters and prediabetes risk

Untraditional lipid parameters were categorized based on quartiles of lipid parameters to verify the robustness of the results and reintroduced into Model 3 to evaluate their relationship with prediabetes (Fig. 2). Relative to the lowest quartile, ORs for prediabetes consistently rose across Q2, Q3, and Q4 of LCI, AIP, non-HDL-C, and RC, exhibiting a progressive increase. Notably, Q3 of AC, CRI-I, CRI-II, and RC/HDL-C ratio exhibited the highest OR values. Fig. 3 illustrates the nonlinear relationship between lipid parameters and prediabetes, except for TC, evident after fitting with smoothing splines (P for nonlinearity < 0.05). Saturation effect points were computed for each parameter to assess the dose–response relationship between lipid parameters and prediabetes risk. Particularly, when AIP was ≤ 0.524, a substantial increase in prediabetes risk was observed with increasing AIP. Conversely, when CRI-II was > 2.059, a gradual decrease in prediabetes risk was observed with increasing CRI-II (Additional file 3: Table S3).

### Performance of lipid parameters in predicting prediabetes

**Fig. 4:**
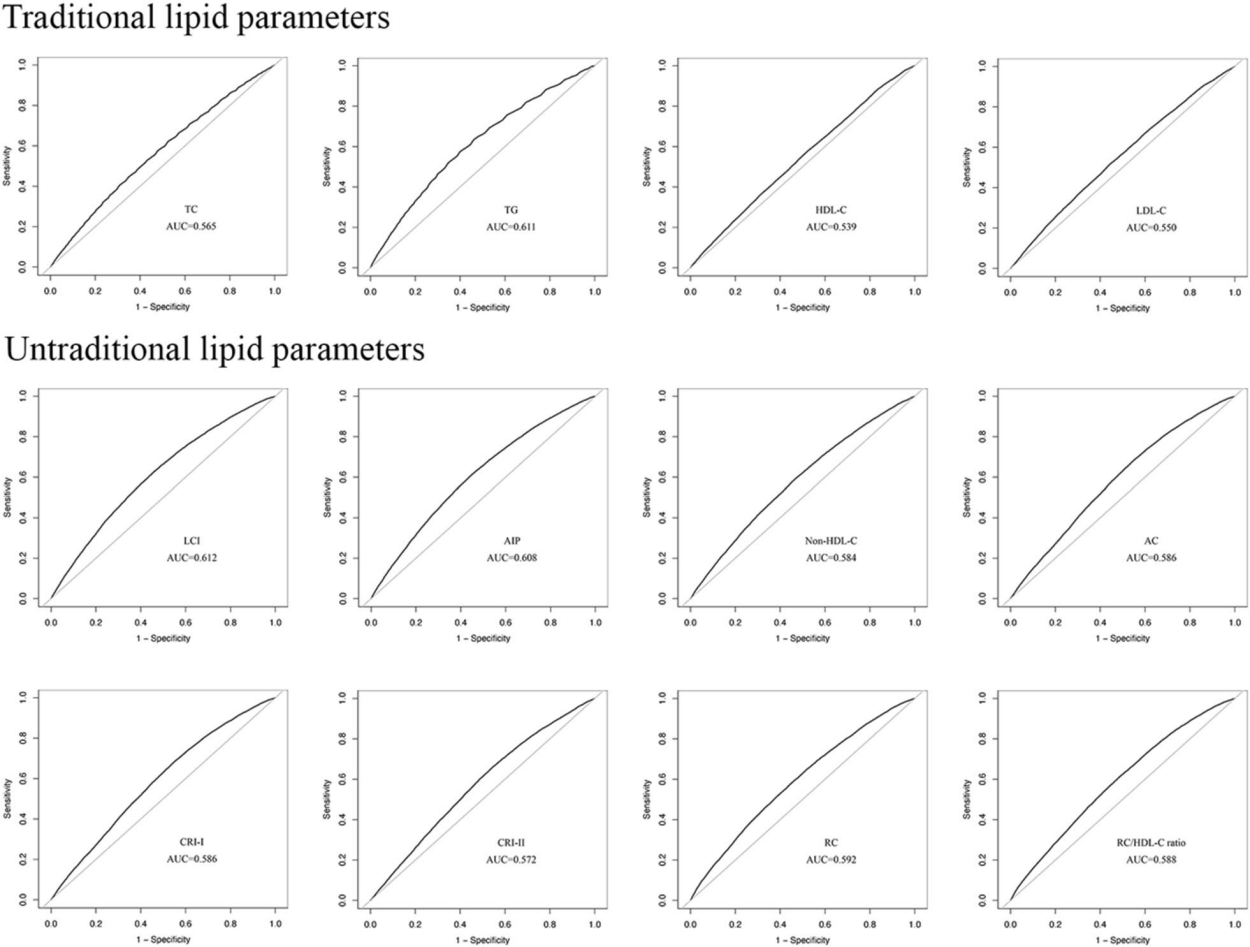
ROC curve analysis of the lipid parameters in predicting prediabetes

ROC curve analysis compared the accuracy of lipid parameters in identifying prediabetes (Fig. 4, Table 5). Remarkably, the AUC for all 12 lipid parameters exceeded 0.5, indicating their utility in prediabetes identification. Relatively, the recognition ability of untraditional lipid parameters for prediabetes surpassed that of TC, HDL-C, and LDL-C. Among the untraditional lipid parameters, LCI exhibited superior recognition ability for prediabetes, exhibiting an optimal critical value of 10.656 and an AUC was 0.612 (0.607–0.617), with a specificity of 0.560 and sensitivity of 0.608.

**Table 5:** The AUC, best threshold, sensitivity, and specificity of lipid parameters in identifying prediabetes

Variables	AUC	95%CI low	95%CI up	Best threshold	Specificity	Sensitivity
TC	0.565	0.560	0.571	4.695	0.522	0.576
TG	0.611	0.606	0.617	1.195	0.600	0.575
HDL-C	0.539	0.534	0.545	1.375	0.477	0.578
LDL-C	0.550	0.545	0.555	2.755	0.559	0.514
LCI	0.612	0.607	0.617	10.656	0.560	0.608
AIP	0.608	0.603	0.613	− 0.088	0.550	0.614
Non-HDL- C	0.584	0.578	0.589	3.290	0.527	0.597
AC	0.586	0.581	0.592	2.260	0.451	0.682
CRI-I	0.586	0.581	0.592	3.260	0.451	0.682
CRI-II	0.572	0.566	0.577	1.876	0.455	0.660
RC	0.592	0.587	0.598	0.585	0.539	0.593
RC/HDL-C ratio	0.588	0.583	0.594	0.415	0.519	0.607

*TC* total cholesterol, *TG* triglyceride, *HDL-C* high-density lipoprotein cholesterol, *LDL-C* low-density lipoprotein cholesterol, *LCI* lipoprotein combine index, *AIP* atherogenic index of plasma, *AC* atherogenic coefficient, *CRI-I* Castelli’s index-I, *CRI-II* Castelli’s index-II, *RC* remnant cholesterol, *AUC* area under the receiver operating characteristic curve, *CI* confidence interval

### Stratified analyses

**Fig. 5:**
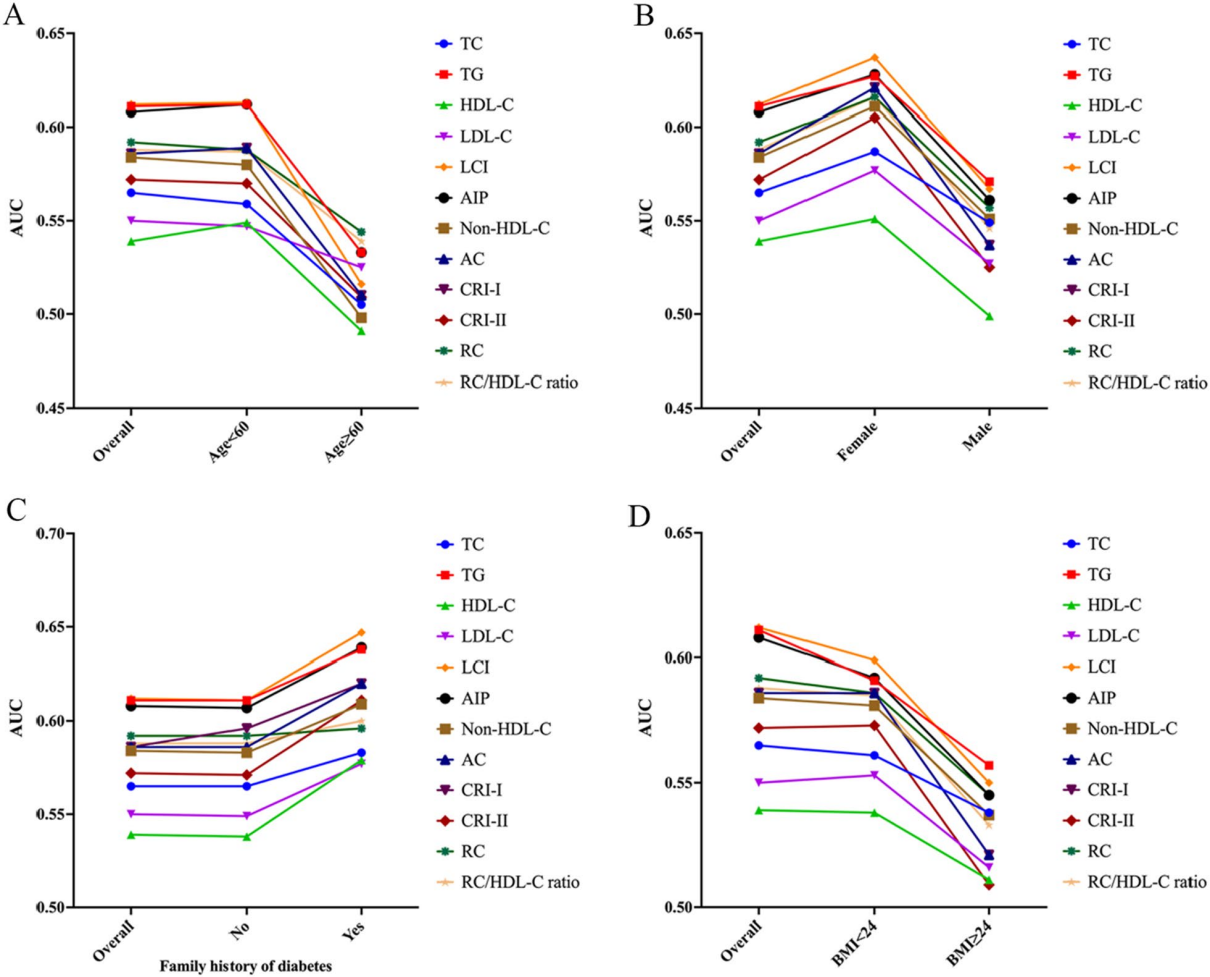
The AUC of lipid parameters in stratified analysis by age (**A**), sex (**B**), family history of diabetes (**C**) and BMI (**D**)

A stratified analysis by age, gender, BMI, and family history of diabetes was conducted to evaluate lipid parameters' ability to discern various populations (Fig. 5). In populations with individuals aged < 60 years, females, those with a BMI of < 24 kg/m^2^, and individuals with a family history of diabetes, stronger associations between lipid parameters and prediabetes risk were observed. Furthermore, the predictive efficacy of untraditional lipid parameters for prediabetes risk slightly surpassed that of traditional lipid parameters. Notably, among the untraditional lipid parameters, LCI emerged as the most reliable predictor for prediabetes. Across diverse stratified analyses, AUCs of all lipid parameters remained within a stable fluctuation range, indicating consistent performance across different population subsets.

## Discussion

This study comprehensively evaluated the correlation and diagnostic significance of 12 lipid parameters concerning prediabetes. The main findings are as follows: (1) Individuals with prediabetes exhibited significantly higher lipid parameter levels compared to those without prediabetes. (2) Lipid parameters demonstrated close associations with METS-IR and CDRS. (3) Following adjustment for confounding factors, traditional lipid parameters highlighted TG as an independent prediabetes risk factor, whereas HDL-C and LDL-C appeared potentially protective. Conversely, untraditional lipid parameters, excluding CRI-II, emerged as independent prediabetes risk factors. Categorizing lipid parameters upheld the robustness of these results. (4) A nonlinear dose–response relationship between lipid parameters and prediabetes risk was observed. (5) In general, the predictive efficacy of untraditional lipid parameters for prediabetes surpassed that of traditional lipid parameters. Notably, LCI emerged as the optimal predictor of prediabetes, irrespective of age, gender, BMI, and family history of diabetes. To sum up, our study underscores that elevated lipid parameters, especially untraditional lipid parameters, could serve as alternative markers of IR and aid in predicting prediabetes in primary clinical settings.

Indeed, abnormalities in blood lipid levels contribute to inflammation, endoplasmic reticulum stress, and lipid toxicity, all culminating in IR [24, 25]. Extensive research has probed the relationship between traditional lipid parameters—HDL-C, TC, LDL-C, and TG—and diabetes, with a consensus that elevated TG and decreased HDL-C significantly increase diabetes risk [26, 27]. However, research on untraditional lipid parameters remains nascent. A recent South Korean national health examination study noted a significant association between elevated TG/HDL-C ratio and increased risk of new-onset diabetes in both sexes [11]. Another nationally representative cross-sectional study highlighted an inverse L-shaped association between AIP and IR, as well as a J-shaped association with diabetes [28]. However, most studies focus solely on individual lipid parameters, lacking comprehensive evaluation and comparison. The optimal untraditional lipid indicators and their critical values for identifying prediabetes remain elusive. Addressing this gap, the current study comprehensively evaluated 12 lipid parameters concerning prediabetes occurrence. Our findings corroborate existing research, further underscoring the heightened predictive value of untraditional lipid parameters, notably LCI, surpassing traditional lipid parameters.

Evidence supports hypertriglyceridemia as a prevalent dyslipidemia characteristic in patients with prediabetes [7]. Elevated TG levels result in increased free fatty acids (FFAs), promoting alterations in pancreatic α cell insulin signaling and excessive glucagon secretion, leading to IR [29]. Conversely, IR exacerbates TG levels by inhibiting TG lipolysis, thereby increasing FFAs in the liver, and reducing HDL-C through decreased apolipoprotein A-I expression, necessary for HDL-C synthesis [30]. The relationship between TG and IR forms a causal relationship, promoting the "vicious circle" of diabetes development. In the current study, TG emerged as the most influential factor associated with prediabetes among all traditional lipid parameters. Similarly, multiple studies have confirmed that fibrates effectively intervene in lipid toxicity to alleviate peripheral tissue IR and pancreatic islet β cell dysfunction, thereby aiding in mitigating the progression of prediabetes [31–33].

While LDL-C is typically viewed as an initiator of cardiovascular disease, its impact on diabetes development presents a contentious issue. In our study, an increase in LDL-C (≥ 2.16 mmol/L) showed a potential for reducing prediabetes risk (OR 0.920, 95% CI 0.889–0.953). This finding, though counterintuitive, aligns with emerging evidence suggesting a protective role for LDL-C. Reports indicate that individuals with familial hypercholesterolemia, characterized by high LDL-C levels, exhibit a lower diabetes prevalence than unaffected relatives [34, 35]. One hypothesis attributes this to mutations in the LDL receptor gene, which decrease pancreatic β cell uptake of LDL particles, thereby inhibiting cell dysfunction and apoptosis [36]. An open-label randomized clinical trial reported that hemoglobin A1c (HbA1c) levels increased after treatment with ezetimibe [37]. The American Heart Association has also stated that statin therapy might elevate the risk of new diabetes [38]. Moreover, studies exploring lipid genetics propose intricate connections. Swerdlow et al. have proposed that LDL-C lowering alleles at 3-hydroxy-3-methylglutaryl-CoA reductase (HMGCR) are associated with increased body weight and diabetes risk, potentially linked to HMGCR inhibition [39]. A large meta-analysis of genetic association studies assessing the effects of cholesterol-lowering variants in or near Niemann-Pick C1-like 1, HMGCR, and LDL receptor showed an overall increased risk of diabetes with an OR of 1.19–2.42 for every 1 mmol/L reduction in LDL-C [40]. These complexities underscore the intricate relationship between mechanisms leading to LDL-C reduction and metabolic risk. Further research is needed to determine the relationship between LDL-C and prediabetes.

Additionally, our study unveiled a different trend, demonstrating that all lipid parameters hold greater diagnostic significance in young, non-obese, and female individuals experiencing prediabetes onset. This implies heterogeneity in the relationship between lipid parameters and prediabetes based on gender, age, and BMI. Compared to older individuals, younger people often adopt unhealthy eating habits and sedentary lifestyles, marked by high-fat diets and irregular sleep patterns, contributing significantly to IR [41]. A lifestyle intervention study for diabetes prevention showed a 39% reduction in the risk of progression to diabetes during the 30-year follow-up [42]. As age advances, the likelihood of various chronic disease complications and other influencing factors increases, leading to a relatively weaker correlation between lipid parameters and prediabetes. Moreover, 61% of newly diagnosed women with prediabetes in this study were over 45 years old, indicating a predominance of prediabetes occurrence after menopause. Research suggests that decreased ovarian function and imbalanced hormone levels in perimenopausal or postmenopausal women, with a dominance of male hormones, prompt central visceral fat accumulation and abdominal obesity [43–45]. This distinct fat deposition pattern adversely affects glucose metabolism, promoting IR in non-adipose tissues and organs, significantly increasing prediabetes risk in postmenopausal women [46]. In concurrence, studies by Shi et al. echo similar sentiments, illustrating a pronounced association between higher AIP levels and increased prediabetes and diabetes prevalence solely in women, not men [47]. This finding emphasizes the need to develop gender-related risk management strategies to prevent prediabetes.

Our study has some significant advantages. First, data from a large survey encompassing a well-defined Chinese national cohort was used. Second, a comprehensive comparison of 12 lipid indicators against prediabetes was conducted for the first time, calculating the optimal critical value and AUC of these indicators in identifying prediabetes among different populations. This not only enhances our understanding of prediabetes risk factors but also provides new insights for precision medicine. Importantly, rigorous statistical analysis bolsters the reliability of our conclusions.

However, certain limitations warrant acknowledgement. First, due to the observational nature of the study, residual confounding factors, albeit adjusted for significant confounding variables, might persist, requiring cautious interpretation of causal relationships. Second, owing to the prohibitive cost of oral glucose tolerance and HbA1c tests for 685,277 adults, the definition of prediabetes in this study encompassed only impaired fasting glucose, potentially underestimating the true prevalence of prediabetes. Additionally, the absence of data on hyperlipidemia and lipid-lowering therapy hinders stratified analysis to assess the impact of lipid-lowering therapy on prediabetes risk. Moreover, the lack of repeated lipid variable measurements precludes the exploration of lipid parameter fluctuations over time. Lastly, generalizing our findings should be approached cautiously, given regional, racial, and dietary habit disparities.

## Conclusion

This study determined the nonlinear relationship between traditional and untraditional lipid parameters and prediabetes risk. Specifically, among young individuals, women, those with a family history of diabetes, and non-obese individuals, LCI, TG, and AIP exhibited superior predictive values for prediabetes compared to other lipid profiles. These findings help clinicians to implement more personalized prevention strategies effectively.

### Supplementary Information

**Additional file 1: Table S1.** Chinese diabetes risk score (CDRS).

**Additional file 2: Table S2.** Collinearity analysis.

**Additional file 3: Table S3.** The result of the two-piecewise logistic regression model.

## Data Availability

The data of this study can be downloaded from the Dryad public database (https://datadryad.org/stash/dataset/doi:10.5061%2Fdryad.ft8750v).
