# Supplementary material for: Nonlinear relationship between untraditional lipid parameters and the risk of prediabetes: a large retrospective study based on Chinese adults
Source: Cardiovasc Diabetol. 2024 Jan 6;23:12. doi: 10.1186/s12933-023-02103-z (PMC10771669; doi:10.1186/s12933-023-02103-z)
Supplement: Supplementary file 1 — Additional file 1: Table S1. Chinese diabetes risk score (CDRS). [file 12933_2023_2103_MOESM1_ESM.docx]

**Supplementary Table 1**. Chinese diabetes risk score (CDRS)

| Scoring Index | Score | Scoring Index | Score |
| --- | --- | --- | --- |
| Age (years) |  | BMI（kg/m^2^） |  |
| 20~24 | 0 | <22.0 | 0 |
| 25~34 | 4 | 22.0~23.9 | 1 |
| 35~39 | 8 | 24.0~29.9 | 3 |
| 40~44 | 11 | ≥30.0 | 5 |
| 45~49 | 12 | Waist circumference (cm) |  |
| 50~54 | 13 | Male <75.0，female <70.0 | 0 |
| 55~59 | 15 | Male 75.0~79.9, female 70.0~74.9 | 3 |
| 60~64 | 16 | Male 80.0~84.9, female 75.0~79.9 | 5 |
| 65~74 | 18 | Male 85.0~89.9, female 80.0~84.9 | 7 |
| SBP (mmHg) |  | Male 90.0~94.9, female 85.0~89.9 | 8 |
| <110 | 0 | Male ≥95.0, female ≥90.0 | 10 |
| 110~119 | 1 | Family history of diabetes  (Parents, siblings, children) |  |
| 120~129 | 3 | No | 0 |
| 130~139 | 6 | Yes | 6 |
| 140~149 | 7 | Gender |  |
| 150~159 | 8 | Female | 0 |
| ≥160 | 10 | Male | 2 |

Abbreviations: SBP, Systolic blood pressure; BMI, Body mass index.
