# Supplementary material for: Nonlinear relationship between untraditional lipid parameters and the risk of prediabetes: a large retrospective study based on Chinese adults
Source: Cardiovasc Diabetol. 2024 Jan 6;23:12. doi: 10.1186/s12933-023-02103-z (PMC10771669; doi:10.1186/s12933-023-02103-z)
Supplement: Supplementary file 2 — Additional file 2: Table S2. Collinearity analysis. [file 12933_2023_2103_MOESM2_ESM.docx]

**Supplementary Table 2**. Collinearity analysis

|  | Unstandardized Coefficients | | Standardized Coefficients | t | Sig. | Collinearity Statistics | |
| --- | --- | --- | --- | --- | --- | --- | --- |
|  | B | Std. Error | Beta |  |  | Tolerance | VIF |
| (Constant) | -0.854 | 0.013 |  | 65.890 | <0.001 |  |  |
| Age | 0.003 | <0.001 | 0.132 | 45.800 | <0.001 | 0.824 | 1.214 |
| Male | -0.131 | 0.002 | -0.224 | -57.536 | <0.001 | 0.447 | 2.238 |
| Smoking status | -0.024 | 0.002 | -0.103 | -13.583 | <0.001 | 0.119 | 8.393 |
| Drinking status | 0.028 | 0.002 | 0.122 | 16.084 | <0.001 | 0.119 | 8.433 |
| Family history of diabetes | 0.031 | 0.005 | 0.016 | 5.909 | <0.001 | 0.977 | 1.023 |
| BMI | 0.028 | <0.001 | 0.305 | 100.897 | <0.001 | 0.742 | 1.347 |
| SBP | <0.001 | <0.001 | -0.017 | -4.276 | <0.001 | 0.455 | 2.198 |
| DBP | 0.002 | <0.001 | 0.087 | 23.171 | <0.001 | 0.485 | 2.061 |
| FPG | 0.022 | 0.002 | 0.035 | 13.310 | <0.001 | 0.960 | 1.041 |
| ALT | 0.002 | <0.001 | 0.128 | 38.809 | <0.001 | 0.624 | 1.601 |
| AST | -0.001 | <0.001 | -0.031 | -9.954 | <0.001 | 0.713 | 1.403 |
| Scr | 0.001 | <0.001 | 0.030 | 7.796 | <0.001 | 0.470 | 2.130 |
| BUN | -0.024 | 0.001 | -0.094 | -33.234 | <0.001 | 0.854 | 1.172 |

Abbreviations: BMI body mass index, SBP systolic blood pressure, DBP diastolic blood pressure, FPG fasting plasma glucose, ALT alanine aminotransferase, AST aspartate aminotransferase, Scr serum creatinine, BUN blood urea nitrogen, VIF variance inflation factor.
