# Supplementary material for: Nonlinear relationship between untraditional lipid parameters and the risk of prediabetes: a large retrospective study based on Chinese adults
Source: Cardiovasc Diabetol. 2024 Jan 6;23:12. doi: 10.1186/s12933-023-02103-z (PMC10771669; doi:10.1186/s12933-023-02103-z)
Supplement: Supplementary file 3 — Additional file 3: Table S3. The result of the two-piecewise logistic regression model. [file 12933_2023_2103_MOESM3_ESM.docx]

**Supplementary Table 3**. The result of the two-piecewise logistic regression model

| Variables | Turning point (K) | <K segment effect 1 | >K segment effect 2 | The difference between the effect of 2 and 1 | P for log-likelihood ratio test |
| --- | --- | --- | --- | --- | --- |
| TC | 6.290 | 1.028(1.001, 1.056),  P=0.044 | 0.937(0.846, 1.037),  P=0.206 | 0.911(0.814, 1.020),  P=0.106 | 0.101 |
| TG | 2.400 | 1.138(1.095, 1.183),  P=0.261 | 1.014(0.984, 1.044),  P=0.366 | 0.891(0.842, 0.942),  P<0.001 | <0.001 |
| HDL-C | 1.640 | 1.076(0.980, 1.182),  P=0.123 | 0.465(0.368, 0.587),  P<0.001 | 0.432(0.326, 0.571),  P<0.001 | <0.001 |
| LDL-C | 2.160 | 1.330(1.127, 1.570),  P<0.001 | 0.920(0.889, 0.953),  P<0.001 | 0.692(0.578, 0.828),  P<0.001 | <0.001 |
| LCI | 3.244 | 1.222(1.096, 1.362),  P=0.261 | 1.002(1.001, 1.003),  P=0.002 | 0.820(0.736, 0.914),  P<0.001 | <0.001 |
| AIP | -0.524 | 10.924(2.913, 40.970),  P<0.001 | 1.278(1.180, 1.386),  P<0.001 | 0.117(0.031, 0.446),  P=0.002 | <0.001 |
| Non-HDL-C | 4.910 | 1.046(1.017, 1.076),  P=0.002 | 0.930(0.838, 1.033),  P=0.175 | 0.890(0.792, 0.999),  P=0.049 | 0.045 |
| AC | 2.503 | 1.211(1.134, 1.292),  P<0.001 | 0.979(0.953, 1.006),  P=0.134 | 0.809(0.747, 0.876),  P<0.001 | 0.001 |
| CRI-I | 3.503 | 1.211(1.134, 1.292),  P<0.001 | 0.979(0.953, 1.006),  P=0.134 | 0.809(0.747, 0.876),  P<0.001 | <0.001 |
| CRI-II | 2.059 | 1.147(1.057, 1.246),  P=0.001 | 0.932(0.895, 0.970),  P<0.001 | 0.812(0.732, 0.901),  P<0.001 | <0.001 |
| RC | 0.350 | 2.942(2.240, 3.864),  P<0.001 | 1.157(1.099, 1.219),  P<0.001 | 0.393(0.294, 0.527),  P<0.001 | <0.001 |
| RC/HDL-C ratio | 0.325 | 3.972(3.040, 5.189),  P=0.001 | 1.059(1.006, 1.115),  P=0.029 | 0.267(0.200, 0.355),  P<0.001 | <0.001 |

Abbreviations: TC total cholesterol, TG triglyceride, HDL-C high-density lipoprotein cholesterol, LDL-C low-density lipoprotein cholesterol, LCI lipoprotein combine index, AIP atherogenic index of plasma, AC atherogenic coefficient, CRI-I Castelli’s index-I, CRI-II Castelli’s index-II, RC remnant cholesterol.
